# A map of neurofilament light chain species in brain and cerebrospinal fluid and alterations in Alzheimer’s disease

**DOI:** 10.1093/braincomms/fcac045

**Published:** 2022-02-22

**Authors:** Melissa M. Budelier, Yingxin He, Nicolas R. Barthelemy, Hong Jiang, Yan Li, Ethan Park, Rachel L. Henson, Suzanne E. Schindler, David M. Holtzman, Randall J. Bateman

**Affiliations:** 1 Department of Neurology, Washington University School of Medicine, St Louis, MO, USA; 2 Department of Pathology and Immunology, Washington University School of Medicine, St Louis, MO, USA; 3 Hope Center for Neurological Disorders, Washington University School of Medicine, St Louis, MO, USA; 4 Charles F. and Joanne Knight Alzheimer’s Disease Research Center, Washington University School of Medicine, St Louis, MO, USA; 5 Department of Biostatistics, Washington University School of Medicine, St Louis, MO, USA

**Keywords:** Neurofilament light, immunoprecipitation-mass spectrometry, Alzheimer’s disease, neurodegeneration

## Abstract

Neurofilament light is a well-established marker of both acute and chronic neuronal damage and is increased in multiple neurodegenerative diseases. However, the protein is not well characterized in brain tissue or body fluids, and it is unknown what neurofilament light species are detected by commercial assays and whether additional species exist. We developed an immunoprecipitation-mass spectrometry assay using custom antibodies targeting various neurofilament light domains, including antibodies targeting Coil 1A/1B of the rod domain (HJ30.13), Coil 2B of the rod domain (HJ30.4) and the tail region (HJ30.11). We utilized our assay to characterize neurofilament light in brain tissue and CSF of individuals with Alzheimer’s disease dementia and healthy controls. We then validated a quantitative version of our assay and measured neurofilament light concentrations using both our quantitative immunoprecipitation-mass spectrometry assay and the commercially available immunoassay from Uman diagnostics in individuals with and without Alzheimer’s disease dementia. Our validation cohort included CSF samples from 30 symptomatic amyloid-positive participants, 16 asymptomatic amyloid-positive participants, 10 symptomatic amyloid-negative participants and 25 amyloid-negative controls. We identified at least three major neurofilament light species in CSF, including N-terminal and C-terminal truncations, and a C-terminal fragment containing the tail domain. No full-length neurofilament light was identified in CSF. This contrasts with brain tissue, which contained mostly full-length neurofilament and a C-terminal tail domain fragment. We observed an increase in neurofilament light concentrations in individuals with Alzheimer’s disease compared with healthy controls, with larger differences for some neurofilament light species than for others. The largest differences were observed for neurofilament light fragments including NfL165 (in Coil 1B), NfL324 (in Coil 2B) and NfL530 (in the C-terminal tail domain). The Uman immunoassay correlated most with NfL324. This study provides a comprehensive evaluation of neurofilament light in brain and CSF and enables future investigations of neurofilament light biology and utility as a biomarker.

## Teaser

Budelier *et al*. provide the first comprehensive evaluation of neurofilament light chain in CSF and brain by mass spectrometry mapping. They developed domain-specific antibodies and a novel immunoprecipitation-mass spectrometry assay with which they identified differential isoforms between CSF and brain samples, including a new C-terminal fragment of neurofilament light.

## Graphical Abstract

**Graphical abstract:**
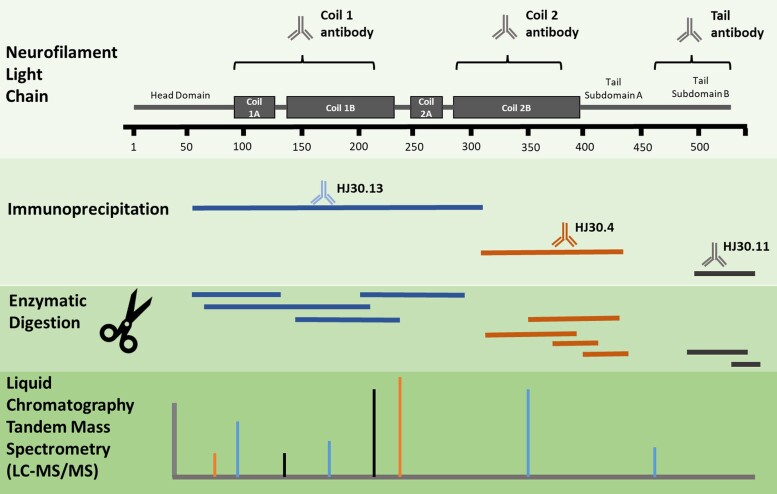

**See Leckey and Zetterberg (https://doi.org/10.1093/braincomms/fcac070) for a scientific commentary on this article.**

## Introduction

Neurofilaments are important structural components of myelinated axons and help to increase axon diameter, allowing for faster nerve conductance. In the CNS, neurofilaments are protein polymers composed of the following four proteins: neurofilament light chain (NfL) and alpha-internexin form the neurofilament core and co-assemble with neurofilament medium chain (NfM) and neurofilament heavy chain (NfH). All four proteins contain conserved rod domains and unique amino-terminal (N-terminal) and carboxyl-terminal (C-terminal) domains.^1^ Of these proteins, NfL is a well-established marker in CSF and plasma of both acute and chronic neuronal damage,^2^ and is increased in Alzheimer’s disease,^3^ frontotemporal dementia,^4^ Parkinson’s disease,^5^ progressive supranuclear palsy,^6^ traumatic brain injury,^7^ multiple sclerosis,^8^ amyotrophic lateral sclerosis^9^ and other neurodegenerative disorders to varying degrees.^10^ While a commercially available immunoassay is utilized for research and has been successful at differentiating neurodegenerative diseases from healthy controls,^11,12^ it is unclear what NfL species the commercial assay detects, and whether additional species exist. The increase of NfL concentrations in multiple diseases that cause neurodegeneration limits its utility in diagnosing and staging disease and potentially monitoring treatment response.^13^

A full profile of NfL species and their relationship to different diseases can help inform our understanding of the pathophysiological processes that generate extracellular NfL as well as potentially identify disease-specific species. Similar to recent discoveries related to specific tau fragments and post-translational modifications (PTMs) as biomarkers,^14–16^ there may be NfL species, such as protein fragments and PTMs, that vary by neurodegenerative process (e.g. inflammatory process versus astrocytic process), by neuron type (e.g. inhibitory versus excitatory, cortical versus subcortical) or mechanism of cell death (e.g. apoptosis or autophagy versus necrosis). Current methods measuring NfL concentrations in biofluids rely on immunoassays. While NfL immunoassays are sensitive, the NfL species targeted by the antibodies used in these assays are not well characterized.

To better understand the diverse forms of NfL present in the brain and biofluids, analytical methods that directly characterize the structure of NfL are needed.^17^ Mass spectrometry offers direct protein characterization, and when combined with purification methods such as immunoprecipitation, provides the analytical specificity needed to fully characterize NfL. We developed antibodies that bind to various regions of NfL and characterized NfL domains recovered by these antibodies using immunoprecipitation-mass spectrometry (IP-MS) in brain tissue and CSF. We demonstrate that most brain NfL is a full-length protein while CSF NfL consists of a mixture of different protein fragments. We then tested the newly identified NfL fragments in a discovery cohort of controls and Alzheimer’s disease samples, and further validated our findings in a confirmation cohort.

## Methods

### Institutional Review Board approval

This study was approved by the Washington University Institutional Review Board. The pooled CSF samples used for assay development were previously obtained from human subjects and stored at −80°C. At the time of initial collection, CSF was centrifuged at 1000× *g* for 10 min to remove cell debris and was immediately frozen at −80°C. Brain samples included previously lysed samples stored at −80°C for assay development, and were from controls without amyloid or tau pathology.^14^ All Alzheimer’s disease samples and control CSF samples were collected during a previous study,^18^ aliquoted and stored at −80°C. Amyloid status was previously defined by PET Pittsburgh Compound-B (PET PIB mean cortical binding potential >0.18 = amyloid-positive) when available, and by CSF Aβ 42/Aβ 40 (concentration ratio of amyloid beta peptide 1–42 divided by amyloid beta peptide 1–40) when PET PIB was not available (CSF Aβ 42/Aβ 40 concentration ratio <0.12 = amyloid-positive).^18^ The validation cohort included CSF samples from 30 symptomatic amyloid-positive participants, 16 asymptomatic amyloid-positive participants, 10 symptomatic amyloid-negative participants and 25 amyloid-negative controls. Participant demographics are shown in Supplementary Table 1.

### Antibody development, screening and characterization

Monoclonal antibodies against recombinant human NfL were generated by immunization of 8-week-old Balb/c3 mice with recombinant NfL protein (rec-NfL) produced in bacteria (head + core, see Supplementary material for amino acid sequence) using complete Freund’s adjuvant (Sigma) as previously described for generation of tau monoclonal antibodies.^19^ For the initial screening of antibodies, supernatants from hybridoma cells were added to 96-well plates coated with rec-NfL. After binding to rec-NfL, the HJ30 series of antibodies were detected by horseradish peroxidase-conjugated anti-mouse IgG. Clones that reacted with rec-NfL and bovine NfL, but not with a negative control protein were grown, sub-cloned and subsequently frozen in liquid nitrogen. Reactivity against human NfL was determined by western blot from the cortex of human brain samples. Twenty-three antibodies underwent further screening and were cross-linked to M270 Epoxy Dynabeads (Invitrogen) according to the manufacturer’s instructions and assessed for their ability to immunoprecipitate full-length rec-NfL and native NfL from pooled CSF used for assay development. Briefly, for rec-NfL, 10 µl of 5 ng/µl rec-NfL in 1% human serum albumin (HSA) were added to 40 µl of 100 mM triethyl ammonium bicarbonate buffer (TEABC). For native NfL, frozen CSF samples were thawed at room temperature, and 450 µl of the thawed CSF was transferred to a new tube. 25 µl of a master mix containing detergent (1% NP-40), chaotropic reagent (5 mM guanidine) and protease inhibitors (Roche complete Protease Inhibitor Cocktail), and 20 µl of 0.5 ng/ml NfL internal standard (ISTD) in 50 mM TEABC was then added. Lys, Arg, ^13^C^15^N labelled full-length rec-NfL (Promise Advanced Proteomics) was used as the ISTD. Both recombinant and native NfL were immunoprecipitated by adding 30 µl of a 30% (i.e. 3 mg/ml) slurry of an antibody-conjugated bead preparation and rotating the sample for 120 min at room temperature. The antibody-conjugated beads were magnetically separated, and the post-IP supernatant was removed. The beads were washed three times in 1 ml of 25 mM TEABC (per wash). The bound NfL was digested on beads with 400 ng MS-grade trypsin/Lys-C (Promega) for 16 h at 37°C. Digests were loaded onto TopTip C18 (Glygen, TT2C18.96), desalted and eluted per the manufacturer’s instructions. The eluants were dried *in vacuo* without heat and stored at −80°C until analysis by liquid chromatography-tandem mass spectrometry (LC-MS/MS) (see the ‘Liquid chromatography-tandem mass spectrometry’ section). Sixteen antibodies recovered full-length recombinant protein (Supplementary Fig. 1). Based on peptide profiles from native NfL immunoprecipitated from pooled CSF, antibodies were determined to have epitopes against the N-terminal portion of the rod domain, the C-terminal portion of the rod domain or the C-terminus of NfL (Supplementary Fig. 2). Antibodies with high recovery and NfL specificity were chosen for each of these NfL domains and used in qualitative and quantitative IP-MS assays. None of the custom antibodies recognized the N-terminus of NfL.

### Qualitative IP-MS method and isoform characterization

A three-step, sequential immunoprecipitation was used to characterize NfL in brain lysate and CSF. Antibodies targeting Coil 1A/1B of the rod domain (HJ30.13), Coil 2B of the rod domain (HJ30.4) and the tail region (HJ30.11) were used. Frozen brain lysates were thawed and a 450 µl aliquot of the thawed brain lysate was diluted 1:1000 with 1% HSA. Frozen CSF samples were thawed at room temperature, and 450 µl of the thawed CSF was transferred to a new 1.6 ml new tube for immunoprecipitation.

Both brain and CSF samples were immunoprecipitated as described above for native CSF using 30 µl of a 30% (i.e. 3 mg/ml) slurry of an antibody-conjugated bead preparation of HJ30.13 (Coil 1A/1B antibody). Washed beads were stored on ice until all samples were ready for on-bead digestion. In the second step, 20 µl of 0.5 ng/ml NfL ISTD in 50 mM TEABC was added, and NfL was immunoprecipitated a second time by adding 30 µl of a 30% (i.e. 3 mg/ml) slurry of an antibody-conjugated bead preparation of HJ30.4 (Coil 2B antibody). The remaining steps were identical to the first immunoprecipitation. Ten nanograms of ISTD in 50 mM TEABC were again added to the post-IP supernatant prior to the third sequential immunoprecipitation, which was performed with HJ30.11 (tail antibody). Bound NfL was digested on beads with 400 ng MS-grade trypsin/Lys-C (Promega) for 16 h at 37°C and samples were extracted as described above.

### Quantitative IP-MS method

To eliminate the need for sequential addition of ISTD, antibodies targeting Coil 1A/1B of the rod domain (HJ30.13), Coil 2B of the rod domain (HJ30.4) and the tail region (HJ30.11) were mixed 1:1:1 to generate an antibody slurry with a final concentration of 10% (i.e. 1 mg/ml) of each antibody. Twenty-five microlitres of a master mix containing detergent (1% NP-40), chaotropic reagent (5 mM guanidine) and protease inhibitors (Roche Complete Protease Inhibitor Cocktail) were added to 96-well plates. Five microlitres of ISTD (0.1 ng/µl in 1% HSA; ISTD solvent and amount optimized for quantitative recovery and assay’s dynamic range) were then added, followed by 450 µl of thawed CSF and 30 µl of the antibody slurry. Immunoprecipitation and on-bead digestion were performed as described above.

Pooled CSF was screened to identify pools with low and high concentrations of NfL. The CSF pools with the lowest (NfL-L1) and highest (NfL-L2) NfL concentrations were selected and used to determine the assay’s linear range. NfL-L2 CSF was serially diluted with NfL-L1 CSF to generate an 8-point curve with 100, 50, 25, 12.5, 6.25, 3.13 and 1.56% of NfL-L2. NfL was immunoprecipitated as described above, in triplicate. The N14/N15 ratios were determined for each of the six peptides in the quantitative method, and the average N14/N15 ratios of the replicates were plotted against %NfL-L2 and linear regression was performed. All six peptides showed good linearity across the tested NfL concentrations, with *R*^2^ ≥ 0.988 (Supplementary Fig. 3). Average % coefficient of variation for each peptide across the linear range was 8–12% (Supplementary Table 2).

### Liquid chromatography-tandem mass spectrometry

Extracted digests were reconstituted with 25 µl of 0.1% formic acid/0% acetonitrile (ACN). A 4.5 µl aliquot of each digest was then injected into nano-Acquity LC for MS analysis. The nano-Acquity LC (Waters Corporation, Milford, MA, USA) was fitted with HSS T3 75 μm × 100 μm, 1.8 μm column and a flow rate of 0.5 μl/min of a gradient of solutions A and B was used to separate the peptides. Solution A was composed of 0.1% formic acid in MS-grade water and solution B was composed of 0.1% formic acid in ACN. Samples were analysed in positive ion mode, with a spray voltage of 2200 V and ion transfer tube temperature of 275°C. Data were collected with parallel reaction monitoring (PRM) for endogenous (N14) and isotopically labelled (Lys, Arg: ^13^C ^15^N) peptides. Tryptic peptides specific to NfL were identified via the Blast search, and those with good ionization were included in the qualitative PRM, designed to optimize sequence coverage. The quantitative method was optimized for assay precision, and multiplexing was reduced to the analysis of six NfL peptides across various NfL domains and their corresponding ISTDs (Supplementary Table 3).

### Data analysis

Data were extracted using Skyline software (MacCoss Laboratory, University of Washington, WA, USA) and exported for further analysis. Peptide trace graphs, amyloid +/− group comparisons and correlation scatterplots were generated using GraphPad Prism version 8.3.0. The associations among NfL peptides, other biomarkers and cognitive/clinical measures were evaluated using Spearman’s correlations. The confidence intervals and *P* values for the correlations were based on Fisher’s *r*-to-*z* transformation. *t*-tests were used to compare amyloid-positive and amyloid-negative groups. Data were log-transformed prior to *t*-tests to account for the right skew. *R* software version 4.0.4 was used for statistical analysis.

### Qualitative discovery cohort

All CSF samples were collected previously^18^ and stored at −80°C. Ten total samples, four Alzheimer’s disease [Clinical Dementia Rating (CDR) 0.5–1, amyloid PET-positive] and six control (CDR 0, amyloid PET-negative), were sequentially immunoprecipitated using HJ30.13, followed by HJ30.4, followed by HJ30.l1. Samples were processed using sequential IP-MS as described above.

### Quantitative validation cohort

The validation cohort consisted of 81 CSF samples previously collected from individuals with Alzheimer’s disease dementia (amyloid-positive, CDR 0–2), non-Alzheimer’s disease dementia (amyloid-negative, CDR 0.5–1) and healthy controls (amyloid-negative, CDR 0).^18^ Amyloid positivity was previously determined by CSF Aβ 42/Aβ 40.^18^ For each CSF sample, six NfL peptides, corresponding to four different domains of NfL (Coil 1A, Coil 1B, Coil 2B and Tail), were measured using the quantitative IP-MS method described above. NfL was also measured via commercial ELISA kit (Uman Diagnostics) according to the manufacturer’s specifications. Briefly, for ELISA measurement, CSF samples were thawed on wet ice and vortexed. Samples were then diluted 2× with the provided sample diluent in a 96-well pre-plate and mixed prior to transferring to the assay plate.

To determine the relationship of soluble NfL species to Alzheimer’s disease clinical, cognitive, imaging and biomarker measures, correlation analysis was performed between each NfL region (IP-MS) and previously obtained biomarker data. The following measures were evaluated: age, CDR-global and CDR Sum of Boxes (CDR-SB), Mini-Mental State Exam (MMSE), amyloid plaque imaging (PET PIB), CSF Aβ 42/Aβ 40,^18^ CSF total tau (t-tau), CSF ptau181 and ptau181/tau181, CSF ptau205 and ptau205/tau205, and CSF ptau217 and ptau217/tau217.^15,20^

### Data and antibody availability

R scripts and data are available upon request. Antibodies will also be made available upon reasonable request, as resources allow.

## Results

### CSF contains multiple NfL species

**Figure 1:**
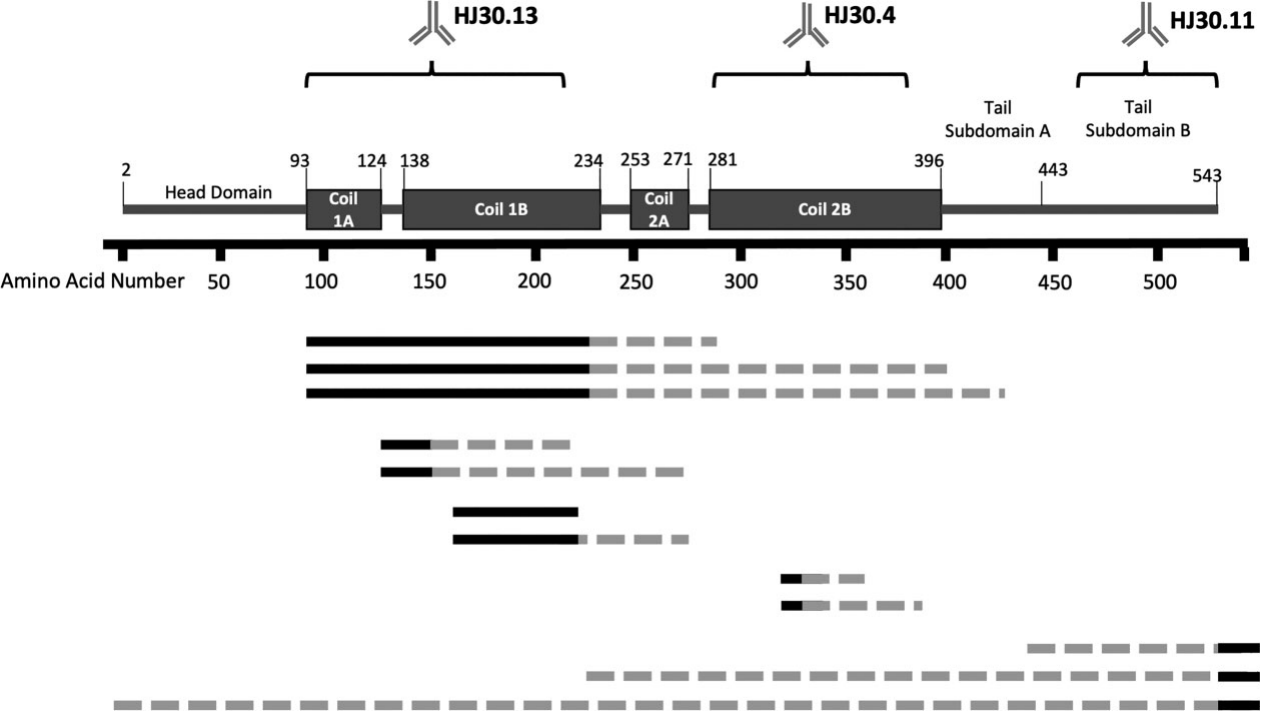
**Map of neurofilament light species indicates that CSF NfL exists as multiple fragment species.** Antibodies targeting various domains of NfL used for immunoprecipitation, coupled with mass spectrometry analysis, enabled identification of multiple NfL species in CSF. Light dotted lines represent potential fragments in NfL species identification, while dark solid lines represent identified fragment species. NfL species were identified using 23 different custom antibodies and data used to determine NfL species are shown in Supplementary Fig. 2.

**Figure 2:**
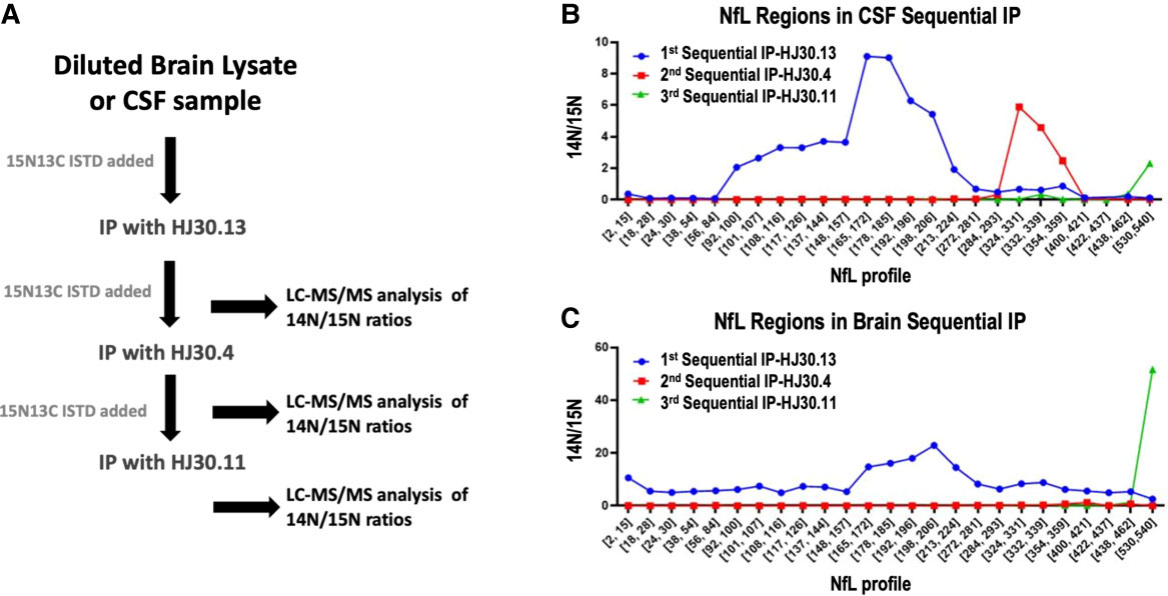
**Brain contains two main NfL species, whereas CSF has at least three main NfL species.** Experimental method for sequential IP-MS/MS assay purifying and identifying at least three NfL fragment species (**A**). Sequential NfL IP from pooled CSF (*n* = 1) indicates three main NfL domains: a mid-domain region from NfL93 to NfL224, another region from NfL324 to NfL359 and a C-terminal region at NfL530 (**B**) and brain cortex lysate (*n* = 1) showing full-length NfL from NfL2 to NfL540, with a C-terminal peptide at NfL 530 (**C**). The blue line depicts peptides identified following the first IP with HJ30.13, the red line depicts peptides identified during the second IP with HJ30.4 and the green line represents peptides identified during the third IP with HJ30.11.

Twenty-three monoclonal antibodies were generated against NfL and evaluated for their ability to immunoprecipitate full-length rec-NfL, NfL from brain lysate and NfL from pooled CSF (see the ‘Methods’ section). Antibodies were characterized by the NfL domain they targeted, their IP-efficiency and their specificity. Representative antibodies for each NfL domain were selected and used for further assay development. Using antibodies targeting various NfL domains, we determined that multiple NfL species exist in CSF (Fig. 1). To better elucidate the NfL species in CSF, pooled CSF samples were sequentially immunoprecipitated starting with an antibody targeting the Coil 1A/1B [approximately amino acids (aa)93–252, HJ30.13], followed by an antibody targeting the Coil 2B (approximately aa272–396, HJ30.4) and finally with an antibody targeting the C-terminus of the tail region (aa520–550, HJ30.11). Based on these protein profiles, we identified a minimum of three major NfL fragment species in CSF, though it is likely that multiple variations of these species exist. These include two different N-terminal and C-terminal truncations containing rod domains enriched by HJ30.13 (aa92 through at least aa224, with possible variations extending through aa360) and HJ30.4 (aa324 through aa360), as well as a C-terminal fragment containing the tail of NfL (enriched by HJ30.11, containing aa530 through at least aa540). No N-terminal fragments were recovered and full-length NfL was not present in quantifiable concentrations in CSF (Fig. 2A and B).

### Brain NfL is mostly full length and a C-terminal fragment

In contrast to the highly fragmented protein in CSF, brain tissue homogenate contained mostly full-length NfL. To determine if any truncated species were also present in brain, we performed the same sequential immunoprecipitation on human brain tissue. While most brain NfL appeared to be full length, we also observed a C-terminal fragment of tail subdomain B containing at least amino acids 530–540 (Fig. 2A and C), similar to the fragment identified in CSF. A fragment containing aa165–224 appears to be enriched by HJ30.13. No additional NfL fragments were enriched in the brain during the second IP (HJ30.4, Coil 2B of the rod domain).

### NfL species are increased in individuals with Alzheimer’s disease compared with healthy controls

**Figure 3:**
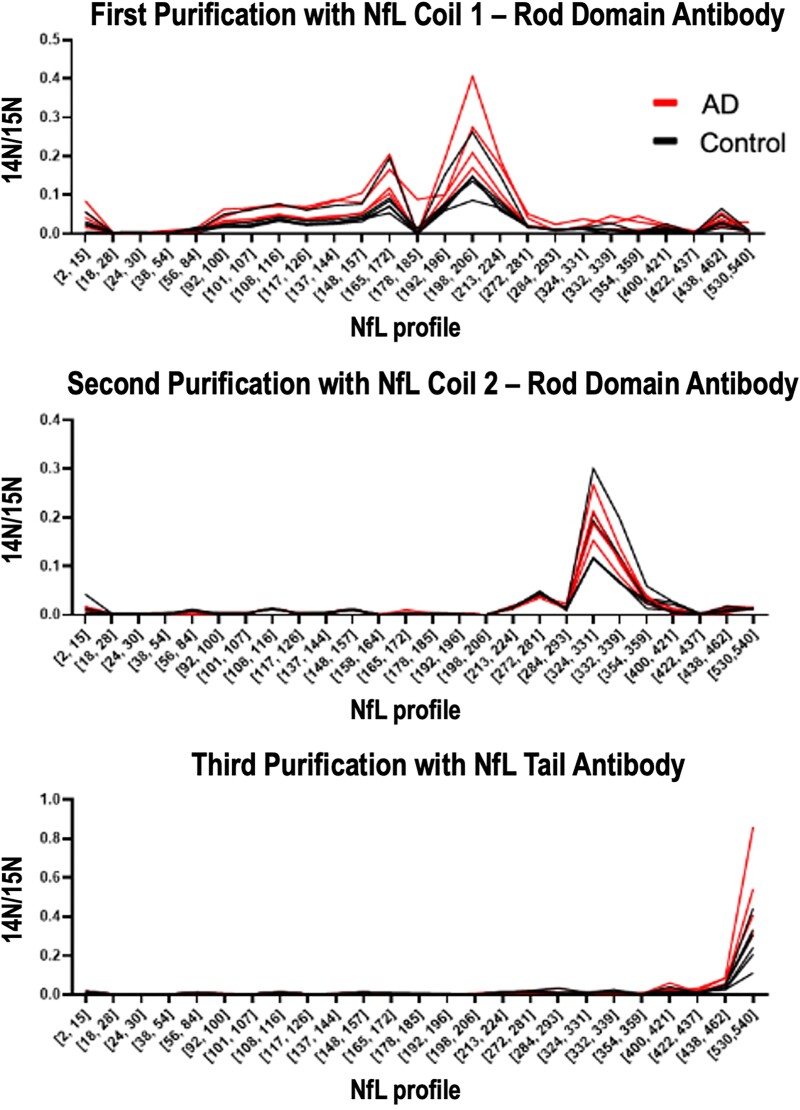
**NfL species are increased in Alzheimer’s disease CSF compared with healthy controls.** Sequential IP-MS of the three main CSF NfL species identifies increased NfL levels in Alzheimer’s disease dementia (*n* = 4) compared with controls (*n* = 6) for each main species. Red lines represent relative amounts of NfL species for individuals with Alzheimer’s disease dementia as determined by the presence of amyloid plaques by PET and very mild dementia (CDR = 0.5) and black lines represent healthy age-matched controls (CDR = 0).

The sequential IP-MS method initially tested on experimental, pooled CSF was repeated on CSF samples from a discovery cohort of Alzheimer’s disease dementia (*n* = 4) and healthy controls (*n* = 6). The peptides observed in both clinical groups were similar to those observed in the pooled CSF, but there were increased amounts of the three major NfL species in Alzheimer’s disease compared with controls (Fig. 3). Additionally, some peptides appeared better than others at differentiating Alzheimer’s disease and control samples. The most prominent difference was observed for the NfL530 [neurofilament light chain tryptic peptide VEGAGEEQAAK(NfL amino acids 530–540)] in the C-terminal tail and tryptic peptide GADEAALAR, within Coil 1B of the rod domain.

To better compare CSF NfL species in Alzheimer’s disease, non-Alzheimer’s disease dementia and healthy controls, we developed a quantitative NfL assay to reliably measure select regions across multiple NfL species. To improve precision, we reduced multiplexing in our quantitative method to measure six peptides across the various NfL domains and their corresponding internal standards. We then applied the IP-MS quantitative method to measure specific CSF NfL species in a validation cohort of 81 Alzheimer’s disease and control samples (30 amyloid-positive, CDR > 0; 16 amyloid-positive, CDR = 0; 10 amyloid-negative, CDR > 0; 25 amyloid-negative, CDR = 0; Supplementary Table 1). For comparison, CSF NfL concentrations were also quantified using the widely used NfL immunoassay from Uman Diagnostics.

**Figure 4:**
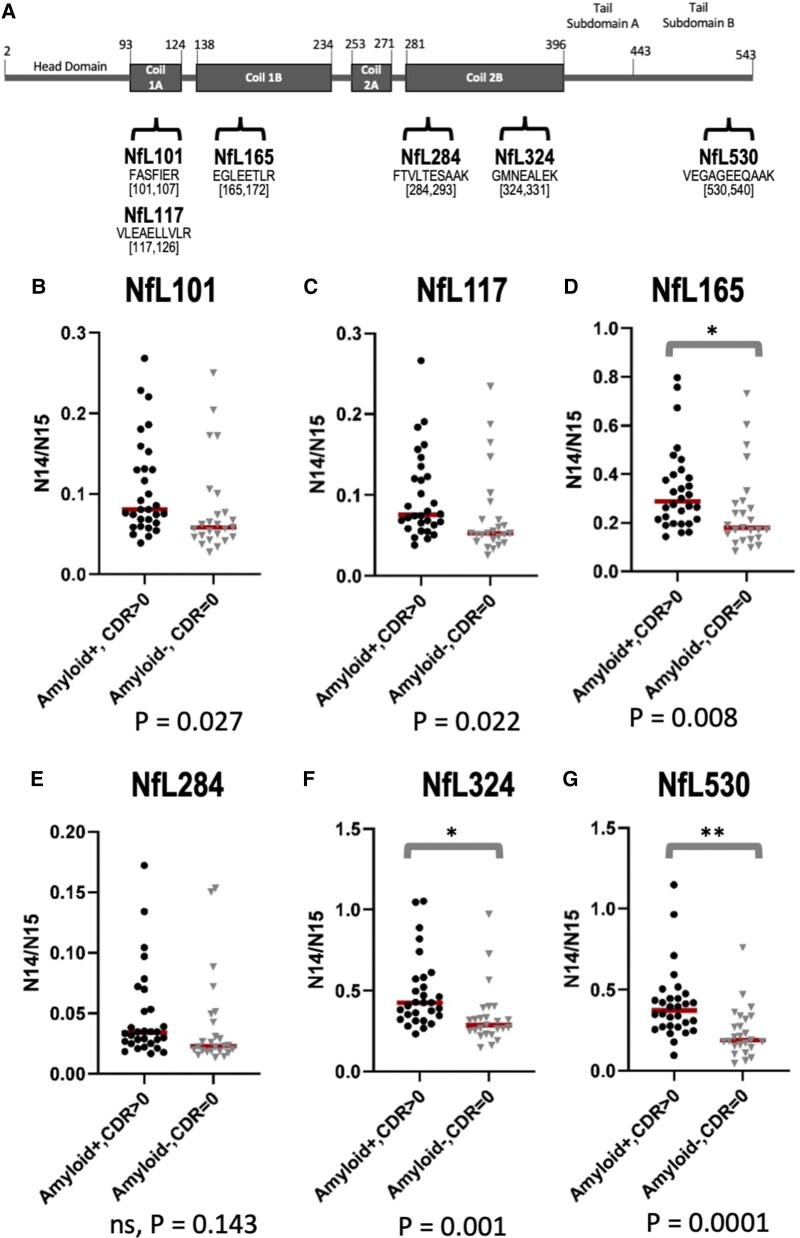
**Validation cohort confirms increased NfL324 and NfL530 in Alzheimer’s disease compared with healthy controls.** Schematic showing NfL map and location of peptides in quantitative IP-MS method (**A**). Comparison of NfL peptides between symptomatic Alzheimer’s disease participants (*n* = 30; amyloid-positive, CDR > 0) and healthy controls (*n* = 25; amyloid-negative, CDR = 0) for Coil 1A and 1B regions NfL101 (**B**), NfL117 (**C**) and NfL165 (**D**) show non-significant increased trends in Alzheimer’s disease, no difference in Coil 2B NfL284 region (**E**) and highly significant increases in NfL324 (**F**), and C-terminal region NfL530 (**G**). Data are right skewed and as such, *t*-tests were performed on log-transformed data. To accurately depict the absolute differences between groups, the *y*-axes were not log-transformed. *Statistical significance at *P* < 0.01; **Statistical significance at *P* < 0.001.

Consistent with sequential IP-MS results of the discovery cohort (Fig. 3), we confirmed increases in NfL concentrations in a confirmation cohort of symptomatic, amyloid-positive individuals (*N* = 30) compared with amyloid-negative healthy controls (*N* = 25) (Fig. 4). Interestingly, the difference between groups was larger for some regions than for others, with the biggest differences observed in Coil 2B of the rod domain (NfL324; GMNEALK, aa324–331) and in the C-terminus of the tail (NfL530; VEGAGEEQAAK, aa530–540). NfL324 [neurofilament light chain tryptic peptide GMNEALEK (NfL amino acids 324–331)] was 1.5-fold increased in Alzheimer’s disease compared with control (*P* = 0.001, Fig. 4F) and NfL530 was 1.7-fold increased (*P* = 0.0001, Fig. 4G). We also measured NfL concentrations for asymptomatic amyloid-positive and symptomatic amyloid-negative individuals and observed an increase in all NfL regions for symptomatic amyloid-negative individuals (Supplementary Fig. 4). The increase in symptomatic amyloid-negative individuals was greater than that for asymptomatic amyloid-positive individuals for all regions except NfL530. We observed a statistically significant increase in all NfL regions for CDR > 0 individuals compared with CDR = 0, independent of amyloid status (Supplementary Fig. 5).

**Figure 5:**
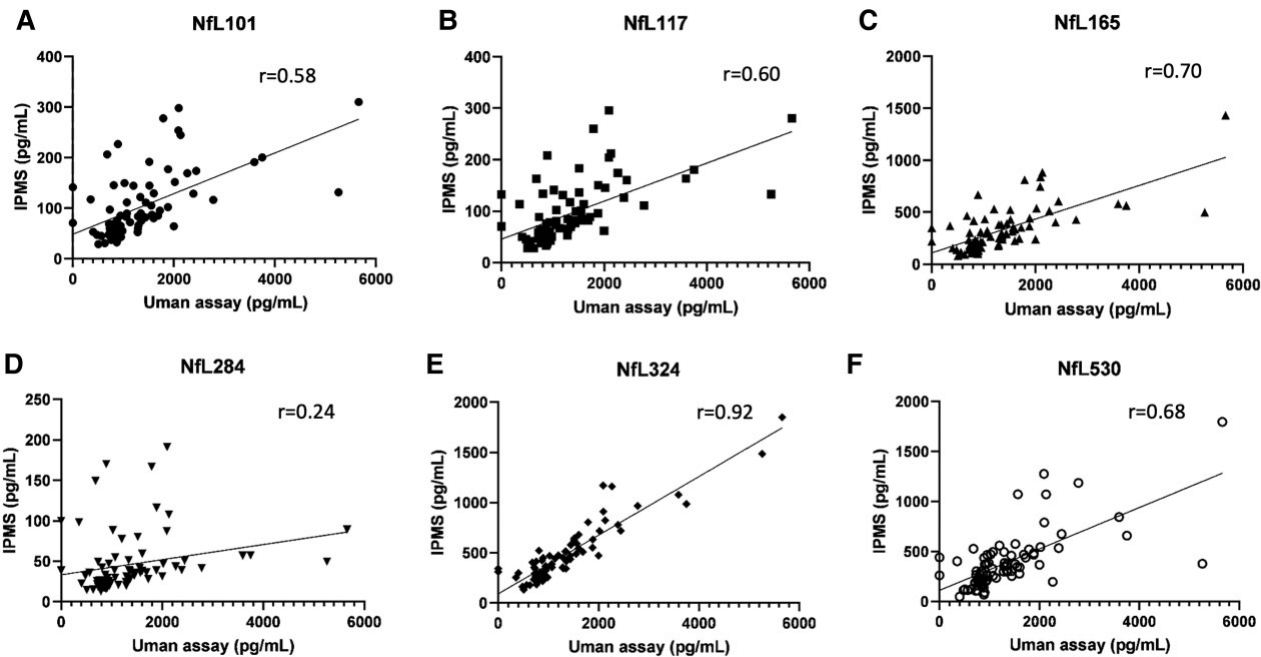
**Correlation between IP-MS and ELISA by NfL species.** Spearman’s correlation between IP-MS and the Uman Diagnostics ELISA results vary by NfL species: NfL101 (**A**), NfL 117 (**B**), NfL 165 (**C**), NfL284 (**D**), NfL 324 (**E**) and NfL530 (**F**). The highest correlation is observed between the ELISA and NfL324.

Using the Uman NfL immunoassay, values were 1.4-fold increased in Alzheimer’s disease compared with controls. The strongest correlation was with NfL324 peptide concentrations (*r* = 0.92) suggesting that the Uman immunoassay targets CSF NfL fragments containing the Coil 2B region. Importantly, correlations between the immunoassay and other investigated peptides were lower for NfL101 [neurofilament light chain tryptic peptide FASFIER (NfL amino acids 101–107)], NfL117 [neurofilament light chain tryptic peptide VLEAELLVLR (NfL amino acids 117–126)], NfL165 [neurofilament light chain tryptic peptide EGLEETLR (NfL amino acids 165–172)]and NfL530 (*r* ranging from 0.58 to 0.70) and no correlation was found with NfL284 [neurofilament light chain tryptic peptide FTVLTESAAK (NfL amino acids 284–293)] (Fig. 5).

**Figure 6:**
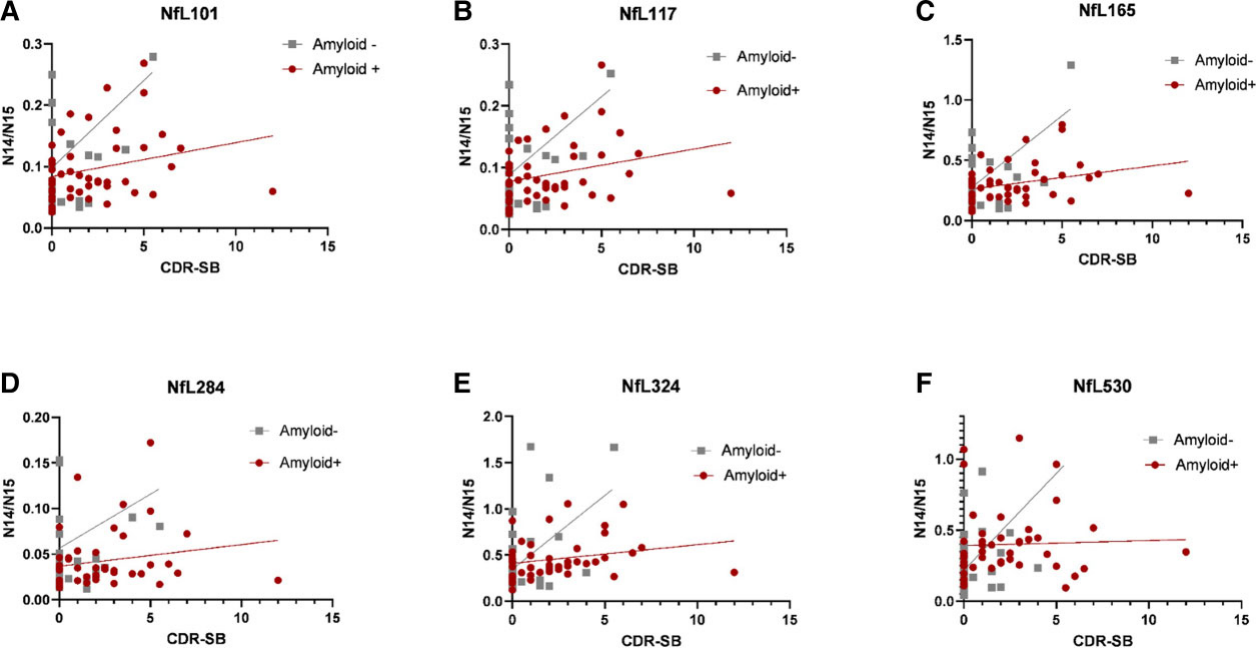
**NfL species correlation with Alzheimer’s disease dementia stage (CDR-SB).** The amount of NfL species are minimally correlated with the stage of dementia severity. The *x*-axis of each graph denotes the CDR-SB, a clinical scale of dementia with CDR-SB 0 is normal, CDR-SB 0.5–6 indicates mild dementia and CDR-SB >6 indicates moderate clinical dementia. The relative amount of NfL species is shown in the *y*-axis as the N14/N15 ratio of the NfL region. Spearman’s correlation and *P*-value were calculated for each group—NfL101: Amyloid+ Spearman *r* = 0.29 (ns, *P* = 0.05), Amyloid– Spearman *r* = 0.18 (ns, *P* = 0.30) (**A**); NfL117: Amyloid+ Spearman *r* = 0.30 (*P* = 0.04), Amyloid– Spearman *r* = 0.18 (ns, *P* = 0.31) (**B**); NfL165: Amyloid+ Spearman *r* = 0.36 (*P* = 0.01), Amyloid– Spearman *r* = 0.19 (ns, *P* = 0.28) (**C**); NfL284: Amyloid+ Spearman *r* = 0.24 (ns, *P* = 0.10), Amyloid– Spearman *r* = 0.31 (ns, *P* = 0.07) (**D**); NfL324: Amyloid+ Spearman *r* = 0.30 (*P* = 0.04), Amyloid– Spearman *r* = 0.16 (ns, *P* = 0.35) (**E**); NfL530: Amyloid+ Spearman *r* = 0.13 (ns, *P* = 0.39), Amyloid– Spearman *r* = 0.25 (ns, *P* = 0.14) (**F**). Participants with amyloid plaques are shown with red circles, and amyloid-negative participants are shown with grey squares. NfL101, NfL117, NfL165 and NfL284 each have one outlier not plotted on the graph but included in calculations of correlation.

As NfL is a marker of general neurodegeneration and not specific to Alzheimer’s disease, we hypothesized that NfL would be increased regardless of the presence of amyloid plaques in those with clinical dementia and neurodegeneration. We evaluated the correlation between CDR-SB (a clinical measure of dementia severity) and NfL species for amyloid-positive and amyloid-negative samples (Fig. 6). While correlation was slightly higher for some NfL species in the amyloid-positive group than the amyloid-negative group (NfL101, NfL117, NfL165 and NfL324), correlation was minimal or low for all NfL species in both groups. Correlation between NfL530 and CDR-SB was not significantly different from 0 for either group.

To form hypotheses about the association of different NfL species with neurodegeneration, we performed Spearman’s correlation analysis between each of the six quantified NfL peptides and additional previously measured biomarkers and clinical measures: general markers of clinical dementia (CDR-SB, MMSE), biomarkers of amyloid plaques (PET PiB, CSF Aβ 42/Aβ 40)^18^ and tau biomarkers (CSF t-tau, CSF phospho-tau immunoassay and mass spectrometry measures of CSF ptau 181, 205 and 217 occupancy),^15,20^Supplementary Fig. 6 and Supplementary Table 4. The goal of this analysis was to form hypotheses about the biology of the different NfL species in general neurodegeneration compared with disease-specific neurodegeneration. The strongest correlations were observed between peptides within Coil 1A (NfL101 and NfL117, *r* = 0.99) and Coil 1B of the rod domain (NfL101 and NfL165, *r* = 0.98; NfL 117 and NfL 165 *r* = 0.98). Peptides in Coil 2B of the rod domain have similar, but slightly lower correlation with the Coil 1A peptides (NfL101 and NfL284, *r* = 0.89; NfL 101 and NfL324, *r* = 0.87; NfL117 and NfL284, *r* = 0.90; NfL117 and NfL324, *r* = 0.88). The correlation between the C-terminal tail peptide and Coil 1A was the lowest among the NfL peptides investigated (NfL101 and NfL530, *r* = 0.75; NfL117 and NfL530, *r* = 0.76). Interestingly, the most C-terminal peptides measured (NfL324 and NfL530) had the highest correlation between disease biomarkers and NfL. The moderate correlation between NfL324 or NfL530 and ptau 181, 205 or 217 ranges from *r* = 0.45 to 0.49. The correlation between the same NfL peptides and t-tau was *r* = 0.42–0.43. Correlation with CSF Aβ 42/Aβ 40 and CSF NfL530 was lower at −0.37 (Fig. 6, Supplementary Table 4).

## Discussion

NfL is increased in the brain and biofluids following neuronal damage and is elevated in multiple neurodegenerative diseases.^2,21^ NfL has been proposed in research studies as a marker of disease severity,^22,23^ and is measured longitudinally in clinical trials to monitor disease progression and response to treatment.^24–26^ While NfL is an established marker of neurodegeneration, to date its measurement has been almost entirely by immunoassay.^17^ Due in part to the limitations of methods used to measure NfL, little is known about the release of NfL from the brain, including the mechanisms of turnover and degradation of NfL, the presence of NfL isoforms in brain and body fluids and the relation of these isoforms to disease.^17^ Here, we have developed antibodies and an analysis strategy to further investigate NfL biology and develop novel assays for different forms of NfL.

Using an IP-MS approach, we discovered there are at least three major NfL truncated species in CSF, and these are increased to varying degrees in Alzheimer’s disease. Furthermore, brain NfL is full length, with a newly identified C-terminal fragment. The major CSF NfL species have different relationships with each other and other Alzheimer’s disease measures. This would indicate NfL truncated species could be differentially secreted in physiologic and neurodegeneration conditions and some of them might be more relevant as biomarkers than others. More studies are needed to investigate the newly identified NfL species. NfL regional levels from NfL Coil 1 domain were highly correlated to each other but different from peptides measured from Coil 2 and C-terminal regions. We found a significant increase of NfL peptides 324 and 530 in symptomatic Alzheimer’s disease CSF supporting these domains might be more relevant as biomarkers. High correlations were observed between NfL324 and the Uman NfL immunoassay and combined with the similar fold increase between Alzheimer’s disease and controls for NfL324 (1.5×) and the ELISA assay (1.4×), suggests that antibodies used by this Uman proprietary assay were likely selected to target these NfL324 regions. This further supports this domain as being relevant in biomarker development. The majority of successful NfL studies used proprietary Uman Diagnostics/Quanterix antibodies to measure NfL concentrations.^17,21,27^ NfL peptides showed modest correlations with CDR, age, and phosphorylated and t-tau, while measures of amyloid PET and MMSE had low correlations. Future studies are needed to characterize the production and turnover of various NfL domains and their relationship to disease states.^14,17,28,29^

The C-terminal fragment of the NfL tail was present in both the brain and CSF. This is particularly interesting, as this fragment, along with the C-terminal portion of Coil 2B, have the largest separation between Alzheimer’s disease and control samples and have the highest correlation with disease-specific clinical markers such as markers of tau pathology, but have a lower correlation with the ELISA than NfL324, suggesting that this is a new isoform that may not be well identified by current ELISA assays.

Another interesting finding was the lack of full-length and N-terminal species in CSF. While our study was able to detect full-length NfL, we do not have an N-terminal NfL antibody, and were unable to determine whether an N-terminal fragment is present in CSF. The N-terminus contains many of NfL’s phosphorylation sites. As such, the development of N-terminal antibodies would be helpful to further characterize NfL in biofluids, and potentially help to identify disease-specific species.

This study is the first comprehensive evaluation of NfL in CSF and brain by mass spectrometry mapping, and this approach will enable future investigations of NfL biology, including comparing patterns of NfL species across neurologic diseases and in response to pathophysiologic processes and identification of novel biomarkers.

## Supplementary Material

fcac045_Supplementary_Data

## Abbreviations

Aβ 42/Aβ 40 =: concentration ratio of amyloid beta peptide 1–42 divided by amyloid beta peptide 1–40
aa =: amino acids
ACN =: acetonitrile
CDR =: Clinical Dementia Rating
CDR-SB =: CDR Sum of Boxes
C-terminal =: carboxyl-terminal
HSA =: human serum albumin
IP-MS =: immunoprecipitation-mass spectrometry
ISTD =: internal standard
LC-MS/MS =: liquid chromatography-tandem mass spectrometry
MMSE =: Mini-Mental State Exam
NfH =: neurofilament heavy chain
NfL =: neurofilament light chain
NfL101 =: neurofilament light chain tryptic peptide FASFIER (NfL amino acids 101–107)
NfL117 =: neurofilament light chain tryptic peptide VLEAELLVLR (NfL amino acids 117–126)
NfL165 =: neurofilament light chain tryptic peptide EGLEETLR (NfL amino acids 165–172)
NfL284 =: neurofilament light chain tryptic peptide FTVLTESAAK (NfL amino acids 284–293)
NfL324 =: neurofilament light chain tryptic peptide GMNEALEK (NfL amino acids 324–331)
NfL530 =: neurofilament light chain tryptic peptide VEGAGEEQAAK(NfL amino acids 530–540)
NfL-L1 =: CSF pool with the lowest NfL concentrations
NfL-L2 =: CSF pool with the highest NfL concentrations
NfM =: neurofilament medium chain
N-terminal =: amino-terminal
PIB =: Pittsburgh Compound-B
PRM =: parallel reaction monitoring
PTMs =: post-translational modifications
rec-NfL =: recombinant NfL protein
TEABC =: triethyl ammonium bicarbonate buffer
t-tau =: total tau
