# Supplementary material for: A map of neurofilament light chain species in brain and cerebrospinal fluid and alterations in Alzheimer’s disease
Source: Brain Commun. 2022 Feb 22;4(2):fcac045. doi: 10.1093/braincomms/fcac045 (PMC8994116; doi:10.1093/braincomms/fcac045)
Supplement: fcac045_Supplementary_Data [file fcac045_supplementary_data.pdf]

Supplemental Material For:

A Map of Neurofilament Light Chain Species in Brain and CSF and Alterations in Alzheimer's Disease

Melissa M Budelier, Yingxin He, Nicolas R. Barthelemy, Hong Jiang, Yan Li, Ethan Park, Rachel L. Henson, Suzanne E. Schindler, David M. Holtzman, Randall J. Bateman

Full length NfL sequence expressed in bacteria to generate HJ30 series antibodies

MSSFSYEPYYSTSYKRRYVETPRVHISSVRSGYSTARSAYSSYSAPVSSS

LSVRRSYSSSSGSLMPSLENLDLSQVAAISNDLKSIRTQEKAQLQDLNDR

FASFIERVHELEQQNKVLEAELLVLRQKHSEPSRFRALYEQEIRDLRLAA

EDATNEKQALQGEREGLEETLRNLQARYEEEVLSREDAEGRLMEARKGAD

EAALARAELEKRIDSLMDEISFLKKVHEEEIAELQAQIQYAQISVEMDVT

KPDLSAALKDIRAQYEKLAAKNMQNAEEWFKSRFTVLTESAAKNTDAVRA

AKDEVSESRRLLKAKTLEIEACRGMNEALEKQLQELEDKQNADISAMQDT

INKLENELRTTKSEMARYLKEYQDLLNVKMALDIEIAAYRKLLEGEETRL

SFTSVGSITSGYSQSSQVFGRSAYGGLQTSSYLMSTRSFPSYYTSHVQEE

QIEVEETIEAAKAEEAKDEPPSEGEAEEEEKDKEEAEEEEAAEEEEAAKE

ESEEAKEEEEGGEGEEGEETKEAEEEEKKVEGAGEEQAAKKKD

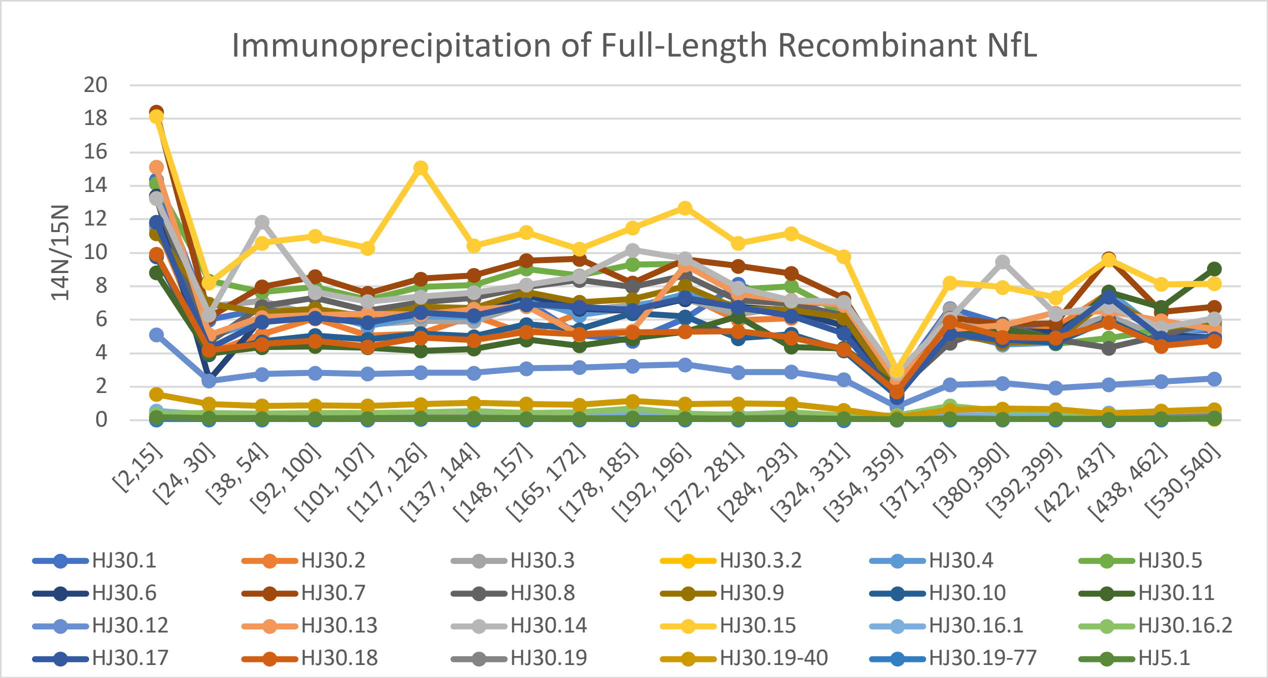

Supplemental Figure 1: Immunoprecipitation of recombinant NfL. Each of 23 NfL antibodies (HJ.30.x) and one negative control antibody against amyloid beta (HJ5.1) were assessed for their ability to immunoprecipitate full length, recombinant NfL. The relative amounts of recombinant NfL recovery are shown (higher N14/N15 = higher recovery of N14. N15 added after IP).

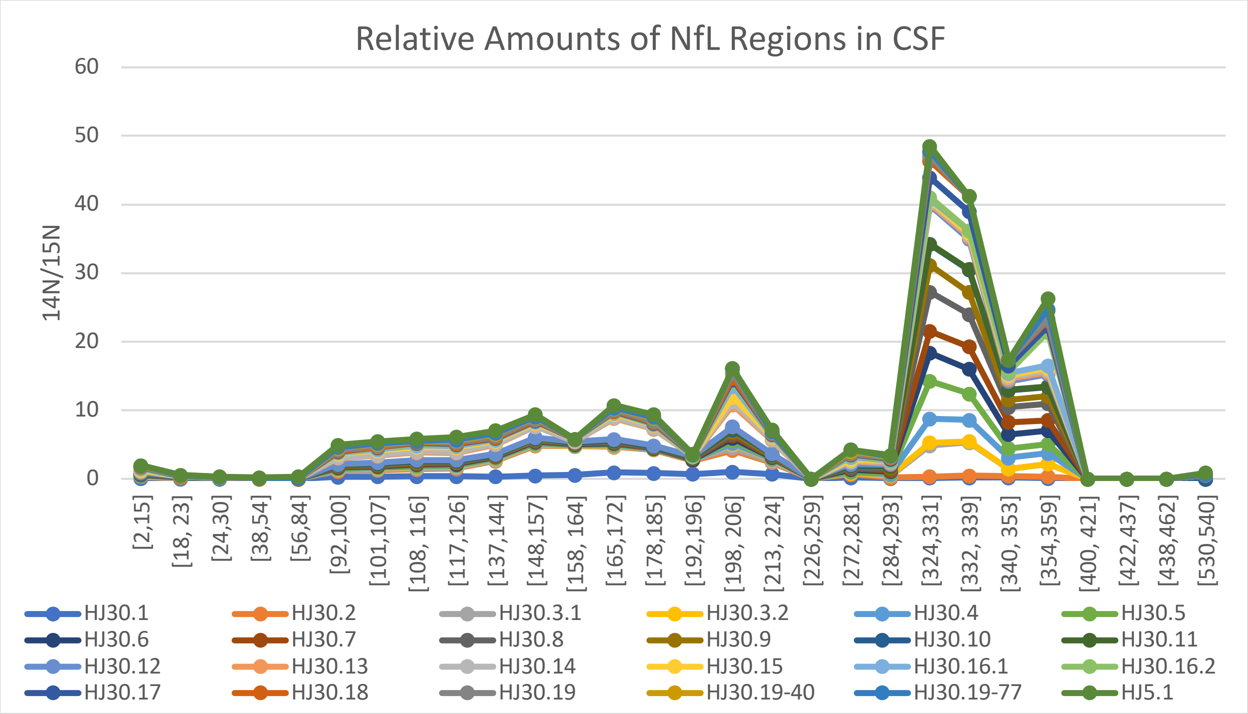

Supplemental Figure 2: Immunoprecipitation of native NfL from pooled CSF. Each of the 23 in-house NfL antibodies (HJ.30.x) and one negative control antibody against amyloid beta (HJ5.1) were assessed for their ability to immunoprecipitate NfL from CSF. Line colors correspond to individual antibodies and are noted in the figure legend. Antibodies appeared to target 3 different regions of NfL (see main text figure 1.)

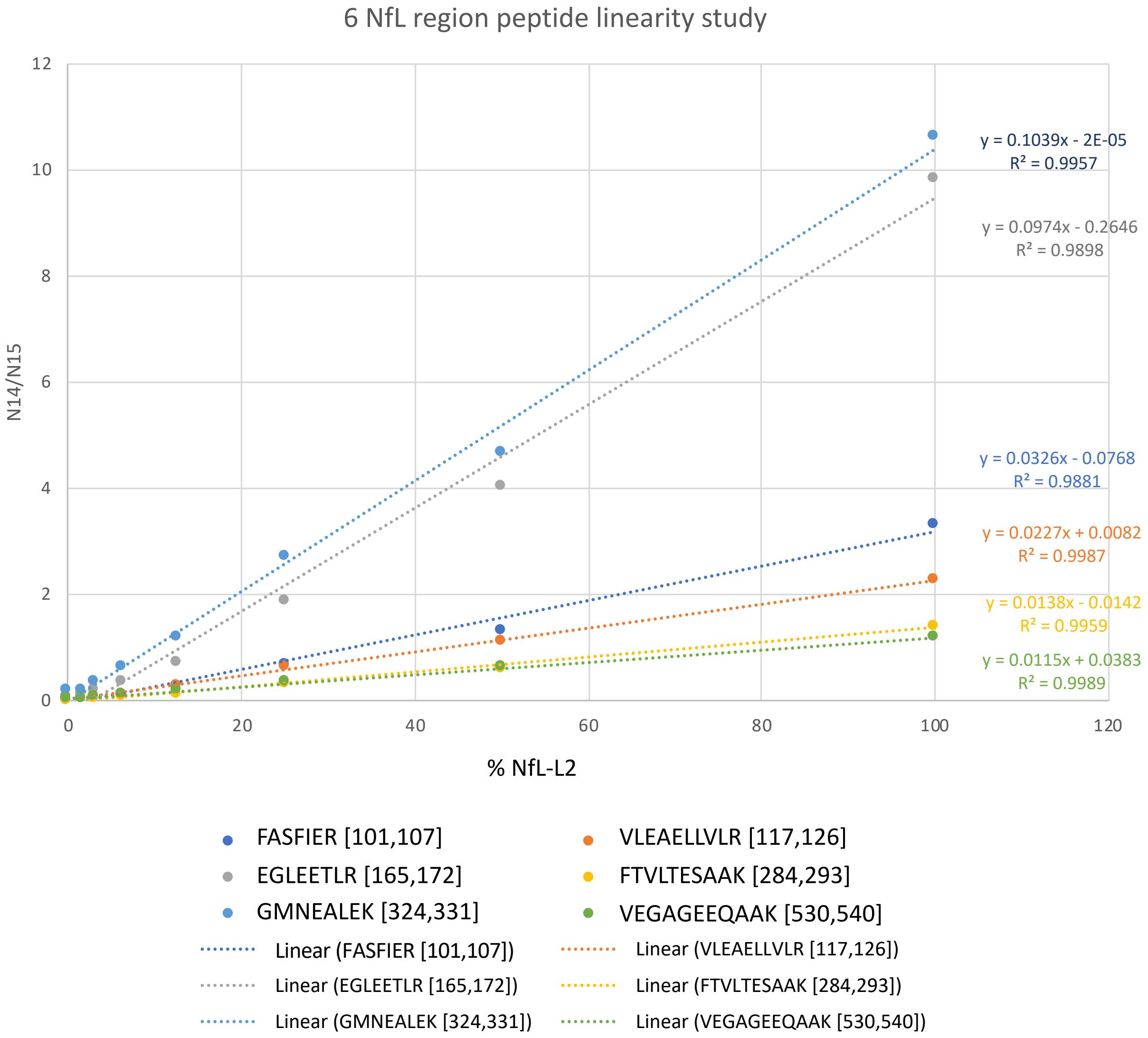

Supplemental Figure 3: Linearity of quantitative (6 peptide) MS method. Quantitation of all 6 peptides is linear within the tested N14/N15 range.

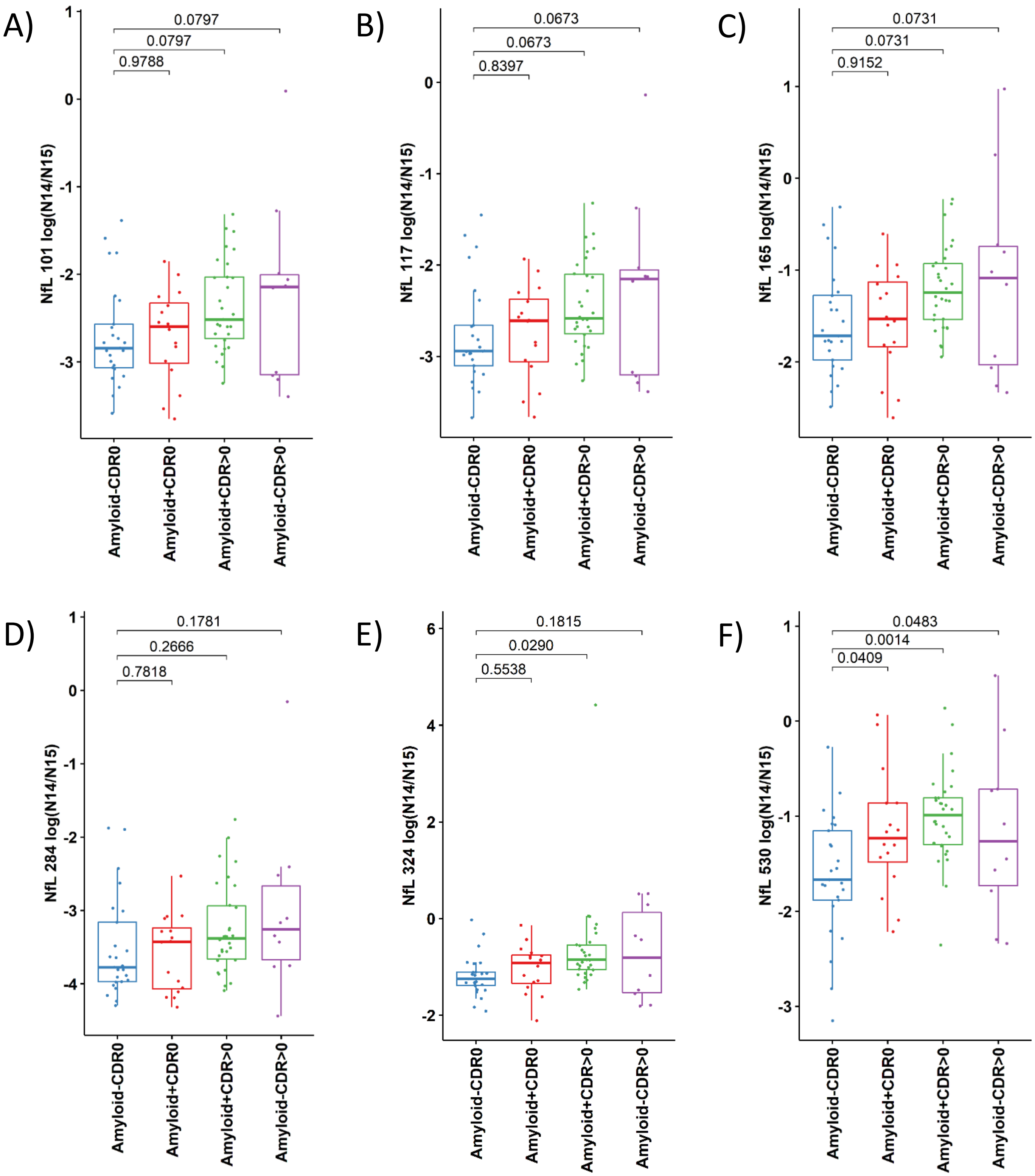
A)
NfL 101 log(N14/N15)
0.0797
0.0797
0.9788
B)
NfL 117 log(N14/N15)
0.0673
0.0673
0.8397
C)
NfL 165 log(N14/N15)
0.0731
0.0731
0.9152
D)
NfL 284 log(N14/N15)
0.1781
0.2666
0.7818
E)
NfL 324 log(N14/N15)
0.1815
0.0290
0.5538
F)
NfL 530 log(N14/N15)
0.0483
0.0014
0.0409
Amyloid-CDR0
Amyloid+CDR0
Amyloid+CDR>0
Amyloid-CDR>0

Supplemental Figure 4: Log transformed NfL concentrations by Amyloid status and CDR. Amyloid- CDR 0 group is used as the reference group and the other three groups were compared to the reference group using two sample t tests. P-values were corrected for multiple comparison using the Benjamini-Hochberg method. NfL concentrations for each of the four groups (Amyloid negative CDR = 0, Amyloid positive CDR = 0, Amyloid positive CDR > 0, Amyloid negative CDR > 0) were compared for NfL 101 (A), NfL 117 (B), NfL 165 (C), NfL 284 (D), NfL 324 (E) and NfL 530 (F).

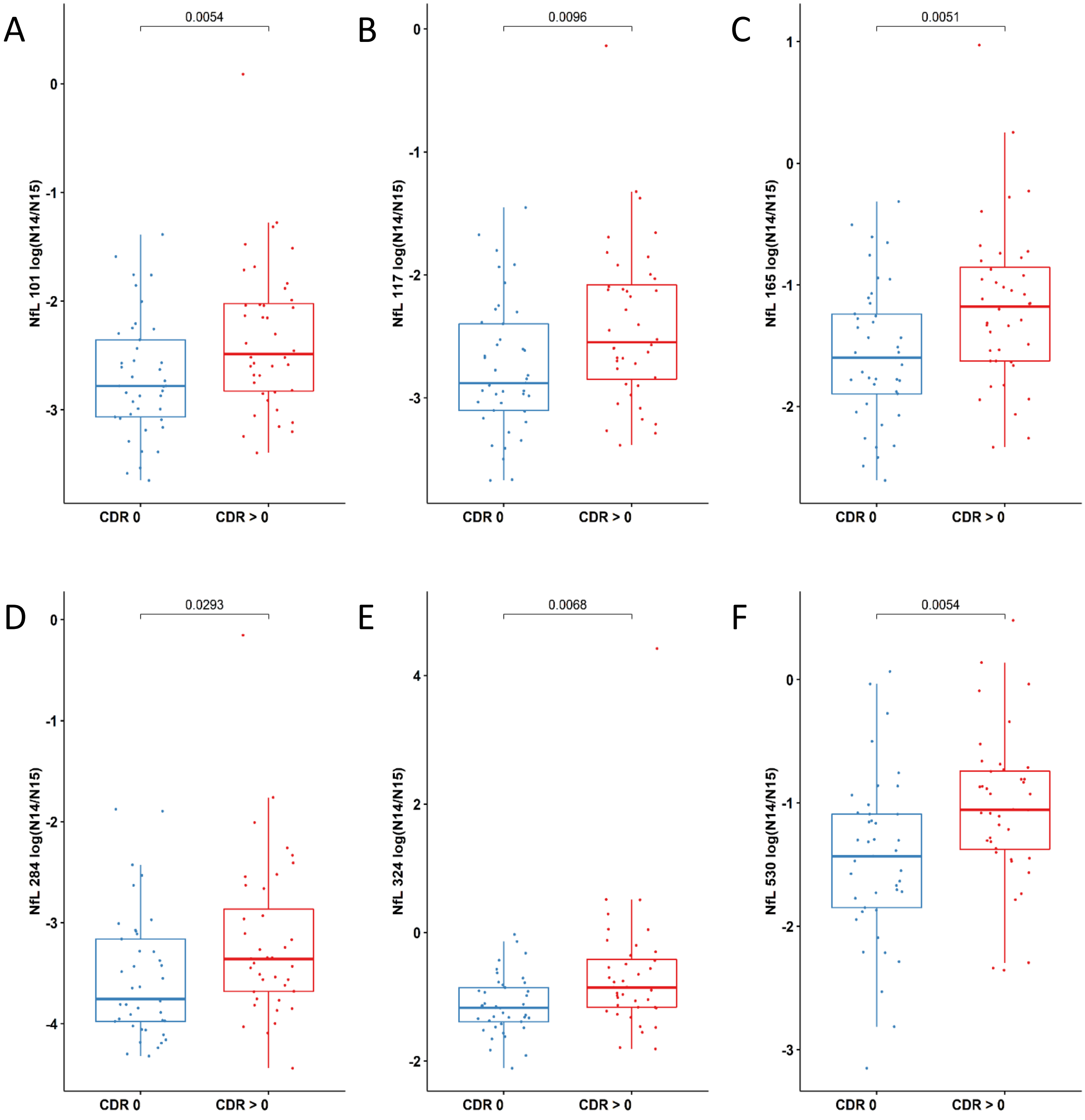

Supplemental Figure 5: Log transformed NfL concentrations by CDR global status. The differences between CDR 0 and CDR > 0 groups were compared using two sample t-tests for NfL 101 (A), NfL 117 (B), NfL 165 (C), NfL 284 (D), NfL 324 (E) and NfL 530 (F).

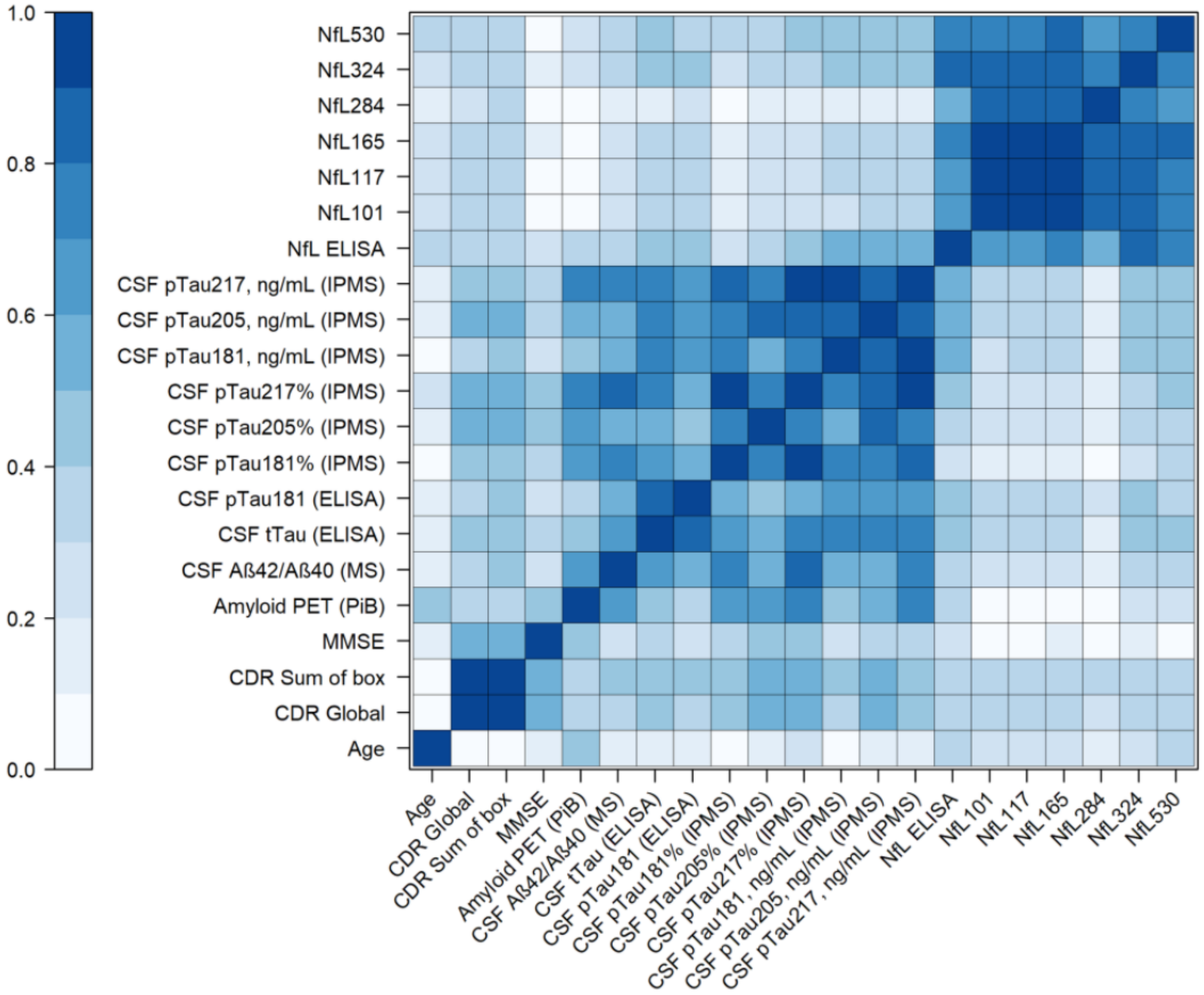

Supplemental Figure 6: Heat map for correlation between NfL species and clinical biomarkers of neurodegeneration, AD, and tau. Map represents Spearman's correlation with darker blue representing stronger correlation and white/light blue representing weak correlation. The strongest correlations of CSF NfL regions were with each other, with NfL324 and NfL530, the C-terminal region, being least correlated with other NfL regions. There were modest correlations between NfL324 or NfL530 and CDR, age, and p-tau and t-tau, while measures of amyloid PET and MMSE had low correlations. Correlations between NfL and tau or ptau were higher for the C-terminal region of NfL (NfL324, NfL530) than for other peptides.

Supplemental Table 1: Demographics of Validation Cohort

Variable	Group			
	**Amyloid+, CDR>0 (n=30)**	**Amyloid+, CDR=0 (n=16)**	**Amyloid-, CDR>0 (n=10)**	**Amyloid-, CDR=0 (n=25)**
Age (mean ± sd)	74.17 ± 6.71	73.43 ± 6.88	74.30 ± 8.22	71.96 ± 5.51
Female (n, %)	9 (30.00%)	9 (56.25%)	2 (20.00%)	14 (56.00%)
APOE4 (n, %)	19 (63.33%)	9 (56.25%)	2 (20.00%)	2 (8.00%)
CDR Sum of boxes (mean ± sd)	3.42 ± 2.39	0.06 ± 0.17	2.15 ± 1.53	0 ± 0
MMSE (mean ± sd)	25.43 ± 3.76	29.25 ± 0.68	27.80 ± 2.25	28.72 ± 1.54

Supplemental Table 2: % CV for IP-MS method across linear range

%NfL-L2	NfL101	NfL117	NfL165	NfL284	NfL324	NfL530
**0**	13	13	19	16	9	13
**1.56**	10	10	11	5	11	8
**3.125**	21	15	22	15	5	7
**6.25**	11	10	8	2	10	18
**12.5**	6	8	8	6	5	6
**25**	4	2	6	2	3	5
**50**	11	5	13	7	8	1
**100**	16	15	6	12	16	21

Supplemental Table 3: Quantitative MS Peptide List

Peptide sequence	Amino acid #	z	Precursor m/z	Fragment ion used for quantitation	Fragment m/z
FASFIER (light)	101-107	2	435.2294	y5+	651.3461
FASFIER (C1315N-ISTD)	101-107	2	440.2336	y5+	661.3543
VLEAELLVLR (light)	117-126	2	577.8608	y8+	942.5619
VLEAELLVLR (C1315N-ISTD)	117-126	2	582.8649	y8+	952.5701
EGLEETLR (light)	165-172	2	473.7456	y4+	518.2933
EGLEETLR (C1315N-ISTD)	165-172	2	478.7498	y4+	528.3016
FTVLTESAAK (light)	284-293	2	533.7926	y8+	818.4618
FTVLTESAAK (C1315N-ISTD)	284-293	2	537.7997	y8+	826.476
GMNEALEK (light)	324-331	2	446.2157	y6+	703.3621
GMNEALEK (C1315N-ISTD)	324-331	2	450.2228	y6+	711.3763
VEGAGEEQAAK (light)	530-540	2	544.7646	y9+	860.4108
VEGAGEEQAAK (C1315N-ISTD)	530-540	2	548.7717	y9+	868.425

Supplementary Table 4: Spearman Correlation table

var1	var2	Correlation	p.value	95% CI
Age	CDR Global	0.07	0.5066	(-0.15, 0.29)
Age	CDR Sum of boxes	0.09	0.4324	(-0.13, 0.3)
Age	MMSE	-0.19	0.0976	(-0.39, 0.03)
Age	Amyloid PET (PiB)	0.47	5.00E-04	(0.22, 0.66)
Age	CSF Aß42/Aß40 (MS)	-0.17	0.1386	(-0.37, 0.05)
Age	CSF tTau (ELISA)	0.19	0.1007	(-0.04, 0.4)
Age	CSF pTau181 (ELISA)	0.18	0.1128	(-0.04, 0.4)
Age	CSF pTau181% (IPMS)	0.07	0.5519	(-0.16, 0.28)
Age	CSF pTau205% (IPMS)	0.12	0.2829	(-0.1, 0.33)
Age	CSF pTau217% (IPMS)	0.22	0.0558	(-0.01, 0.42)
Age	CSF pTau181, ng/mL (IPMS)	0.09	0.4359	(-0.13, 0.3)
Age	CSF pTau205, ng/mL (IPMS)	0.13	0.2544	(-0.09, 0.34)
Age	CSF pTau217, ng/mL (IPMS)	0.2	0.0754	(-0.02, 0.4)
Age	NfL ELISA	0.36	0.0011	(0.15, 0.54)
Age	NfL101	0.24	0.0293	(0.03, 0.44)
Age	NfL117	0.23	0.0411	(0.01, 0.42)
Age	NfL165	0.25	0.0224	(0.04, 0.45)
Age	NfL284	0.19	0.0834	(-0.03, 0.4)
Age	NfL324	0.29	0.0091	(0.07, 0.48)
Age	NfL530	0.38	4.00E-04	(0.18, 0.56)
CDR Global	CDR Sum of boxes	0.96	<0.0001	(0.94, 0.97)
CDR Global	MMSE	-0.56	<0.0001	(-0.69, -0.39)
CDR Global	Amyloid PET (PiB)	0.38	0.0064	(0.11, 0.6)
CDR Global	CSF Aß42/Aß40 (MS)	-0.39	3.00E-04	(-0.56, -0.19)
CDR Global	CSF tTau (ELISA)	0.44	1.00E-04	(0.23, 0.6)
CDR Global	CSF pTau181 (ELISA)	0.39	5.00E-04	(0.18, 0.57)
CDR Global	CSF pTau181% (IPMS)	0.43	1.00E-04	(0.23, 0.6)
CDR Global	CSF pTau205% (IPMS)	0.55	<0.0001	(0.38, 0.69)
CDR Global	CSF pTau217% (IPMS)	0.51	<0.0001	(0.33, 0.66)
CDR Global	CSF pTau181, ng/mL (IPMS)	0.4	2.00E-04	(0.2, 0.57)
CDR Global	CSF pTau205, ng/mL (IPMS)	0.5	<0.0001	(0.32, 0.65)
CDR Global	CSF pTau217, ng/mL (IPMS)	0.47	<0.0001	(0.28, 0.63)
CDR Global	NfL ELISA	0.35	0.0018	(0.14, 0.53)
CDR Global	NfL101	0.3	0.0057	(0.09, 0.49)
CDR Global	NfL117	0.31	0.0049	(0.1, 0.49)
CDR Global	NfL165	0.33	0.0026	(0.12, 0.51)
CDR Global	NfL284	0.27	0.0142	(0.06, 0.46)

CDR Global	NfL324	0.36	0.0012	(0.15, 0.53)
CDR Global	NfL530	0.31	0.005	(0.1, 0.49)
CDR Sum of boxes	MMSE	-0.55	<0.0001	(-0.68, -0.37)
CDR Sum of boxes	Amyloid PET (PiB)	0.34	0.0147	(0.07, 0.57)
CDR Sum of boxes	CSF Aß42/Aß40 (MS)	-0.46	<0.0001	(-0.61, -0.27)
CDR Sum of boxes	CSF tTau (ELISA)	0.48	<0.0001	(0.29, 0.64)
CDR Sum of boxes	CSF pTau181 (ELISA)	0.46	<0.0001	(0.26, 0.62)
CDR Sum of boxes	CSF pTau181% (IPMS)	0.48	<0.0001	(0.29, 0.63)
CDR Sum of boxes	CSF pTau205% (IPMS)	0.57	<0.0001	(0.4, 0.71)
CDR Sum of boxes	CSF pTau217% (IPMS)	0.55	<0.0001	(0.38, 0.69)
CDR Sum of boxes	CSF pTau181, ng/mL (IPMS)	0.43	1.00E-04	(0.23, 0.59)
CDR Sum of boxes	CSF pTau205, ng/mL (IPMS)	0.52	<0.0001	(0.35, 0.67)
CDR Sum of boxes	CSF pTau217, ng/mL (IPMS)	0.5	<0.0001	(0.32, 0.65)
CDR Sum of boxes	NfL ELISA	0.37	9.00E-04	(0.16, 0.54)
CDR Sum of boxes	NfL101	0.34	0.002	(0.13, 0.52)
CDR Sum of boxes	NfL117	0.34	0.0016	(0.14, 0.52)
CDR Sum of boxes	NfL165	0.37	8.00E-04	(0.16, 0.54)
CDR Sum of boxes	NfL284	0.3	0.0059	(0.09, 0.49)
CDR Sum of boxes	NfL324	0.38	4.00E-04	(0.18, 0.56)
CDR Sum of boxes	NfL530	0.33	0.0025	(0.12, 0.51)
MMSE	Amyloid PET (PiB)	-0.43	0.0017	(-0.64, -0.18)
MMSE	CSF Aß42/Aß40 (MS)	0.22	0.0459	(0, 0.42)
MMSE	CSF tTau (ELISA)	-0.31	0.0071	(-0.5, -0.09)
MMSE	CSF pTau181 (ELISA)	-0.25	0.0282	(-0.45, -0.03)
MMSE	CSF pTau181% (IPMS)	-0.38	5.00E-04	(-0.56, -0.18)
MMSE	CSF pTau205% (IPMS)	-0.46	<0.0001	(-0.62, -0.27)
MMSE	CSF pTau217% (IPMS)	-0.46	<0.0001	(-0.62, -0.26)
MMSE	CSF pTau181, ng/mL (IPMS)	-0.28	0.0102	(-0.47, -0.07)
MMSE	CSF pTau205, ng/mL (IPMS)	-0.38	5.00E-04	(-0.55, -0.18)
MMSE	CSF pTau217, ng/mL (IPMS)	-0.37	6.00E-04	(-0.55, -0.17)
MMSE	NfL ELISA	-0.24	0.0332	(-0.44, -0.02)
MMSE	NfL101	-0.1	0.3894	(-0.31, 0.12)
MMSE	NfL117	-0.1	0.3956	(-0.31, 0.13)
MMSE	NfL165	-0.14	0.2266	(-0.34, 0.09)
MMSE	NfL284	-0.03	0.8157	(-0.24, 0.19)
MMSE	NfL324	-0.14	0.231	(-0.34, 0.09)
MMSE	NfL530	-0.06	0.5854	(-0.28, 0.16)
Amyloid PET (PiB)	CSF Aß42/Aß40 (MS)	-0.63	<0.0001	(-0.77, -0.42)
Amyloid PET (PiB)	CSF tTau (ELISA)	0.41	0.0035	(0.14, 0.62)
Amyloid PET (PiB)	CSF pTau181 (ELISA)	0.38	0.0067	(0.11, 0.6)
Amyloid PET (PiB)	CSF pTau181% (IPMS)	0.61	<0.0001	(0.39, 0.76)

Amyloid PET (PiB)	CSF pTau205% (IPMS)	0.61	<0.0001	(0.4, 0.76)
Amyloid PET (PiB)	CSF pTau217% (IPMS)	0.77	<0.0001	(0.63, 0.87)
Amyloid PET (PiB)	CSF pTau181, ng/mL (IPMS)	0.41	0.0028	(0.15, 0.62)
Amyloid PET (PiB)	CSF pTau205, ng/mL (IPMS)	0.54	<0.0001	(0.31, 0.71)
Amyloid PET (PiB)	CSF pTau217, ng/mL (IPMS)	0.72	<0.0001	(0.56, 0.83)
Amyloid PET (PiB)	NfL ELISA	0.31	0.0298	(0.03, 0.54)
Amyloid PET (PiB)	NfL101	0.05	0.7195	(-0.23, 0.33)
Amyloid PET (PiB)	NfL117	0.05	0.7115	(-0.23, 0.33)
Amyloid PET (PiB)	NfL165	0.08	0.5918	(-0.21, 0.35)
Amyloid PET (PiB)	NfL284	0.02	0.9001	(-0.26, 0.3)
Amyloid PET (PiB)	NfL324	0.22	0.1266	(-0.06, 0.47)
Amyloid PET (PiB)	NfL530	0.23	0.1017	(-0.05, 0.48)
CSF Aß42/Aß40 (MS)	CSF tTau (ELISA)	-0.67	<0.0001	(-0.78, -0.53)
CSF Aß42/Aß40 (MS)	CSF pTau181 (ELISA)	-0.6	<0.0001	(-0.73, -0.43)
CSF Aß42/Aß40 (MS)	CSF pTau181% (IPMS)	-0.77	<0.0001	(-0.84, -0.66)
CSF Aß42/Aß40 (MS)	CSF pTau205% (IPMS)	-0.56	<0.0001	(-0.69, -0.38)
CSF Aß42/Aß40 (MS)	CSF pTau217% (IPMS)	-0.81	<0.0001	(-0.88, -0.72)
CSF Aß42/Aß40 (MS)	CSF pTau181, ng/mL (IPMS)	-0.59	<0.0001	(-0.71, -0.42)
CSF Aß42/Aß40 (MS)	CSF pTau205, ng/mL (IPMS)	-0.6	<0.0001	(-0.72, -0.44)
CSF Aß42/Aß40 (MS)	CSF pTau217, ng/mL (IPMS)	-0.76	<0.0001	(-0.84, -0.65)
CSF Aß42/Aß40 (MS)	NfL ELISA	-0.31	0.0054	(-0.5, -0.1)
CSF Aß42/Aß40 (MS)	NfL101	-0.22	0.0466	(-0.42, 0)
CSF Aß42/Aß40 (MS)	NfL117	-0.22	0.0435	(-0.42, -0.01)
CSF Aß42/Aß40 (MS)	NfL165	-0.24	0.0345	(-0.43, -0.02)
CSF Aß42/Aß40 (MS)	NfL284	-0.16	0.1418	(-0.37, 0.06)
CSF Aß42/Aß40 (MS)	NfL324	-0.3	0.0065	(-0.49, -0.09)
CSF Aß42/Aß40 (MS)	NfL530	-0.37	6.00E-04	(-0.55, -0.17)
CSF tTau (ELISA)	CSF pTau181 (ELISA)	0.82	<0.0001	(0.72, 0.88)
CSF tTau (ELISA)	CSF pTau181% (IPMS)	0.68	<0.0001	(0.53, 0.79)
CSF tTau (ELISA)	CSF pTau205% (IPMS)	0.57	<0.0001	(0.39, 0.71)
CSF tTau (ELISA)	CSF pTau217% (IPMS)	0.7	<0.0001	(0.56, 0.8)
CSF tTau (ELISA)	CSF pTau181, ng/mL (IPMS)	0.77	<0.0001	(0.66, 0.85)
CSF tTau (ELISA)	CSF pTau205, ng/mL (IPMS)	0.75	<0.0001	(0.63, 0.84)
CSF tTau (ELISA)	CSF pTau217, ng/mL (IPMS)	0.75	<0.0001	(0.64, 0.84)
CSF tTau (ELISA)	NfL ELISA	0.48	<0.0001	(0.28, 0.64)
CSF tTau (ELISA)	NfL101	0.3	0.008	(0.08, 0.5)
CSF tTau (ELISA)	NfL117	0.31	0.0072	(0.09, 0.5)
CSF tTau (ELISA)	NfL165	0.32	0.0045	(0.11, 0.51)
CSF tTau (ELISA)	NfL284	0.19	0.0995	(-0.04, 0.4)
CSF tTau (ELISA)	NfL324	0.43	1.00E-04	(0.22, 0.6)
CSF tTau (ELISA)	NfL530	0.42	2.00E-04	(0.21, 0.59)

CSF pTau181 (ELISA)	CSF pTau181% (IPMS)	0.58	<0.0001	(0.4, 0.72)
CSF pTau181 (ELISA)	CSF pTau205% (IPMS)	0.42	2.00E-04	(0.21, 0.59)
CSF pTau181 (ELISA)	CSF pTau217% (IPMS)	0.56	<0.0001	(0.37, 0.7)
CSF pTau181 (ELISA)	CSF pTau181, ng/mL (IPMS)	0.68	<0.0001	(0.54, 0.79)
CSF pTau181 (ELISA)	CSF pTau205, ng/mL (IPMS)	0.62	<0.0001	(0.46, 0.74)
CSF pTau181 (ELISA)	CSF pTau217, ng/mL (IPMS)	0.63	<0.0001	(0.47, 0.75)
CSF pTau181 (ELISA)	NfL ELISA	0.45	1.00E-04	(0.25, 0.62)
CSF pTau181 (ELISA)	NfL101	0.34	0.0025	(0.13, 0.53)
CSF pTau181 (ELISA)	NfL117	0.34	0.0026	(0.13, 0.53)
CSF pTau181 (ELISA)	NfL165	0.34	0.0025	(0.13, 0.53)
CSF pTau181 (ELISA)	NfL284	0.25	0.0339	(0.02, 0.45)
CSF pTau181 (ELISA)	NfL324	0.4	4.00E-04	(0.19, 0.58)
CSF pTau181 (ELISA)	NfL530	0.4	4.00E-04	(0.19, 0.57)
CSF pTau181% (IPMS)	CSF pTau205% (IPMS)	0.71	<0.0001	(0.57, 0.8)
CSF pTau181% (IPMS)	CSF pTau217% (IPMS)	0.91	<0.0001	(0.87, 0.94)
CSF pTau181% (IPMS)	CSF pTau181, ng/mL (IPMS)	0.8	<0.0001	(0.7, 0.87)
CSF pTau181% (IPMS)	CSF pTau205, ng/mL (IPMS)	0.79	<0.0001	(0.69, 0.86)
CSF pTau181% (IPMS)	CSF pTau217, ng/mL (IPMS)	0.88	<0.0001	(0.82, 0.92)
CSF pTau181% (IPMS)	NfL ELISA	0.29	0.0106	(0.07, 0.48)
CSF pTau181% (IPMS)	NfL101	0.16	0.1579	(-0.06, 0.37)
CSF pTau181% (IPMS)	NfL117	0.17	0.1345	(-0.05, 0.38)
CSF pTau181% (IPMS)	NfL165	0.17	0.1274	(-0.05, 0.38)
CSF pTau181% (IPMS)	NfL284	0.09	0.4451	(-0.14, 0.3)
CSF pTau181% (IPMS)	NfL324	0.25	0.0276	(0.03, 0.45)
CSF pTau181% (IPMS)	NfL530	0.33	0.0029	(0.12, 0.51)
CSF pTau205% (IPMS)	CSF pTau217% (IPMS)	0.78	<0.0001	(0.68, 0.86)
CSF pTau205% (IPMS)	CSF pTau181, ng/mL (IPMS)	0.55	<0.0001	(0.38, 0.69)
CSF pTau205% (IPMS)	CSF pTau205, ng/mL (IPMS)	0.85	<0.0001	(0.78, 0.9)
CSF pTau205% (IPMS)	CSF pTau217, ng/mL (IPMS)	0.7	<0.0001	(0.57, 0.8)
CSF pTau205% (IPMS)	NfL ELISA	0.37	9.00E-04	(0.16, 0.55)
CSF pTau205% (IPMS)	NfL101	0.22	0.0498	(0, 0.42)
CSF pTau205% (IPMS)	NfL117	0.23	0.0402	(0.01, 0.43)
CSF pTau205% (IPMS)	NfL165	0.26	0.0223	(0.04, 0.45)
CSF pTau205% (IPMS)	NfL284	0.15	0.1792	(-0.07, 0.36)
CSF pTau205% (IPMS)	NfL324	0.33	0.0033	(0.11, 0.51)
CSF pTau205% (IPMS)	NfL530	0.34	0.0024	(0.12, 0.52)
CSF pTau217% (IPMS)	CSF pTau181, ng/mL (IPMS)	0.75	<0.0001	(0.63, 0.83)
CSF pTau217% (IPMS)	CSF pTau205, ng/mL (IPMS)	0.82	<0.0001	(0.73, 0.88)
CSF pTau217% (IPMS)	CSF pTau217, ng/mL (IPMS)	0.94	<0.0001	(0.91, 0.96)
CSF pTau217% (IPMS)	NfL ELISA	0.41	2.00E-04	(0.2, 0.58)
CSF pTau217% (IPMS)	NfL101	0.26	0.0197	(0.04, 0.46)

CSF pTau217% (IPMS)	NfL117	0.27	0.0144	(0.06, 0.47)
CSF pTau217% (IPMS)	NfL165	0.29	0.0104	(0.07, 0.48)
CSF pTau217% (IPMS)	NfL284	0.18	0.1131	(-0.04, 0.39)
CSF pTau217% (IPMS)	NfL324	0.38	6.00E-04	(0.17, 0.55)
CSF pTau217% (IPMS)	NfL530	0.44	<0.0001	(0.25, 0.61)
CSF pTau181, ng/mL (IPMS)	CSF pTau205, ng/mL (IPMS)	0.89	<0.0001	(0.83, 0.92)
CSF pTau181, ng/mL (IPMS)	CSF pTau217, ng/mL (IPMS)	0.91	<0.0001	(0.86, 0.94)
CSF pTau181, ng/mL (IPMS)	NfL ELISA	0.53	<0.0001	(0.35, 0.67)
CSF pTau181, ng/mL (IPMS)	NfL101	0.29	0.0076	(0.08, 0.48)
CSF pTau181, ng/mL (IPMS)	NfL117	0.3	0.0063	(0.09, 0.49)
CSF pTau181, ng/mL (IPMS)	NfL165	0.32	0.0036	(0.11, 0.5)
CSF pTau181, ng/mL (IPMS)	NfL284	0.16	0.1446	(-0.06, 0.37)
CSF pTau181, ng/mL (IPMS)	NfL324	0.45	<0.0001	(0.26, 0.61)
CSF pTau181, ng/mL (IPMS)	NfL530	0.48	<0.0001	(0.3, 0.63)
CSF pTau205, ng/mL (IPMS)	CSF pTau217, ng/mL (IPMS)	0.9	<0.0001	(0.85, 0.93)
CSF pTau205, ng/mL (IPMS)	NfL ELISA	0.53	<0.0001	(0.36, 0.68)
CSF pTau205, ng/mL (IPMS)	NfL101	0.32	0.004	(0.1, 0.5)
CSF pTau205, ng/mL (IPMS)	NfL117	0.32	0.0033	(0.11, 0.51)
CSF pTau205, ng/mL (IPMS)	NfL165	0.35	0.0016	(0.14, 0.52)
CSF pTau205, ng/mL (IPMS)	NfL284	0.19	0.0907	(-0.03, 0.39)
CSF pTau205, ng/mL (IPMS)	NfL324	0.47	<0.0001	(0.28, 0.63)
CSF pTau205, ng/mL (IPMS)	NfL530	0.47	<0.0001	(0.28, 0.62)
CSF pTau217, ng/mL (IPMS)	NfL ELISA	0.51	<0.0001	(0.33, 0.66)
CSF pTau217, ng/mL (IPMS)	NfL101	0.3	0.0061	(0.09, 0.49)
CSF pTau217, ng/mL (IPMS)	NfL117	0.31	0.0046	(0.1, 0.5)
CSF pTau217, ng/mL (IPMS)	NfL165	0.32	0.0031	(0.11, 0.51)
CSF pTau217, ng/mL (IPMS)	NfL284	0.18	0.1179	(-0.04, 0.38)
CSF pTau217, ng/mL (IPMS)	NfL324	0.47	<0.0001	(0.27, 0.62)
CSF pTau217, ng/mL (IPMS)	NfL530	0.49	<0.0001	(0.31, 0.64)
NfL ELISA	NfL101	0.67	<0.0001	(0.53, 0.78)
NfL ELISA	NfL117	0.7	<0.0001	(0.56, 0.8)
NfL ELISA	NfL165	0.72	<0.0001	(0.6, 0.81)
NfL ELISA	NfL284	0.56	<0.0001	(0.38, 0.69)
NfL ELISA	NfL324	0.88	<0.0001	(0.81, 0.92)
NfL ELISA	NfL530	0.72	<0.0001	(0.59, 0.81)
NfL101	NfL117	0.99	<0.0001	(0.99, 1)
NfL101	NfL165	0.98	<0.0001	(0.97, 0.99)
NfL101	NfL284	0.89	<0.0001	(0.84, 0.93)
NfL101	NfL324	0.87	<0.0001	(0.8, 0.91)
NfL101	NfL530	0.75	<0.0001	(0.64, 0.83)
NfL117	NfL165	0.98	<0.0001	(0.97, 0.99)

NfL117	NfL284	0.9	<0.0001	(0.84, 0.93)
NfL117	NfL324	0.88	<0.0001	(0.82, 0.92)
NfL117	NfL530	0.76	<0.0001	(0.65, 0.84)
NfL165	NfL284	0.86	<0.0001	(0.79, 0.91)
NfL165	NfL324	0.88	<0.0001	(0.82, 0.92)
NfL165	NfL530	0.81	<0.0001	(0.71, 0.87)
NfL284	NfL324	0.71	<0.0001	(0.58, 0.8)
NfL284	NfL530	0.67	<0.0001	(0.53, 0.77)
NfL324	NfL530	0.75	<0.0001	(0.63, 0.83)
